# “To teach is to learn twice” Added value of peer learning among medical students during COVID-19 Pandemic

**DOI:** 10.15694/mep.2020.000127.2

**Published:** 2021-06-29

**Authors:** Sara Mohammed Sami Hamad, Shazia Iqbal, Alreuof Mohammed Alothri, Manal Abdullah Ali Alghamadi, Manal Khalid Kamal Ali Elhelow

**Affiliations:** 1 Alfarabi College of Medicine Riyadh; 2 Alfarabi College of Medicine Riyadh

**Keywords:** Peer learning style, Adaption to the COVID-19 Pandemic, Learning strategies during COVID-19, Collaborative learning, Medical student's learning styles

## Abstract

This article was migrated. The article was marked as recommended.

In medical education, peer learning has a significant impact on deeper learning and considered an effective method of collaborative and deeper learning. This article highlights the adjustment of the final year medical students to the peer learning style during the COVID-19 pandemic. It explores the additional benefits of peer learning style and recommend key points that can help medical students to combat the current stressful situation. Adaptation to peer learning strategy may help to overcome this stressful situation and motivate each other to focus on studies. This approach can assist medical students to stay in touch with each other, collaborate, communicate, and boost each other morally. The peer learning style provides an opportunity for students to share thoughts and emotional reactions freely and friendly. This way can help to reduce stress and develop resilience. Students get rapid adaptation to technology-enhanced learning smoothly and effectively by helping each other to learn new skills. The feeling of staying connected with peers during the online sessions significantly augmented the ability to combat the crisis and augment social interactions.

## Introduction

Recently, the COVID-19 pandemic has significantly affected almost all ages, races, and categories of professionals worldwide. Because of the current situation, there is an instinct for transformation to a rapidly changing environment physically, psychologically, and emotionally. This adaptation has effects at personal, institutional, and national levels. We observe a considerable transition in teaching strategies at medical institutions. These amendments have a significant impact on medical students learning styles.

In medical education, peer learning has a substantial impact on deeper learning and considered an effective method of promoting higher-order thinking and metacognition ( Coffman, McConkey, and Colee, 2020). Most of the final year medical students adopted the peer learning style to adjust a new learning paradigm and distance learning through learning management systems. Although few students used to work in pairs and small groups before the pandemic, the significance of this learning style was unrecognized for most students.

## Settings

In Alfarabi college of medicine, the formal teaching strategies are interactive lecturing, problem-based learning, practicals, tutorials, skill lab sessions, and seminars. To avoid the spread of coronavirus, all on-campus teaching activities were suspended. The eruptions of pandemic affected the medical students learning on campus and distressed them more than any other partner of the education cycle. Final year medical students were facing more challenges than another level of students because they have to graduate and secure their career. Because of tough situations, the final year’s medical students adapted to peer learning strategies during March and April 2020.

Since medical campuses were closed and final year medical students made pairs and small groups according to their own choices. Our facilitators encouraged us to study in pairs, small groups, and assigned tasks as teams. The aim was to encourage students to help each other academically, socially, and mentally ( Varalakshmi, 2020; Rastegar *et al.*, 2020). We divided our class into groups, including three to six students in each group. Regular online meetings, sharing ideas on original subjects and topics significantly improved our sense of responsibility for our learning.

In this article, students have reflected on the peer learning experience and mentioned the best practice points for optimal learning during this crisis. The representatives of the peer group conducted seven focused groups online interviews and each group was having four to six students. For analysis, they coded data and identified themes based on grounded theory. The reporters took an iterative method of concurrent data collection and analysis until they reached time saturation. Authors highlighted the change to peer learning style, explored the additional benefits of peer learning style during COVID-19 chaos, and recommended key points that can help medical students to combat the current stressful situation.

## Peer learning beyond collaborative learning

Peer learning improves the learning process through active discussion, sharing ideas, and encourages students toward higher-level thinking through challenging questions ( Elhawary *et al.*, 2019). Students develop their skills to work in groups and collaborate to fulfil the task. This enhances teamwork skills and task executive abilities ( Hunt, Jones, and Carney, 2020). Students learn self-management, time handling skills to boost their energy towards the task ( Zheng and Zhang, 2020). Additionally, it provided them the convenience to learn the skills of conflict resolution during teamwork.

During exams in April, our better achievement during formative evaluations and online written assessments depicted the quality of our learning through this approach. We noticed an improvement in our problem-solving potential through critical thinking by recalling our group discussion. The students’ observation of high performance during formative assessments and comparison of their current results with the previous summative results showed the real value of peer learning.

## Significance of peer learning during COVID-19 Pandemic

**Figure 1.**
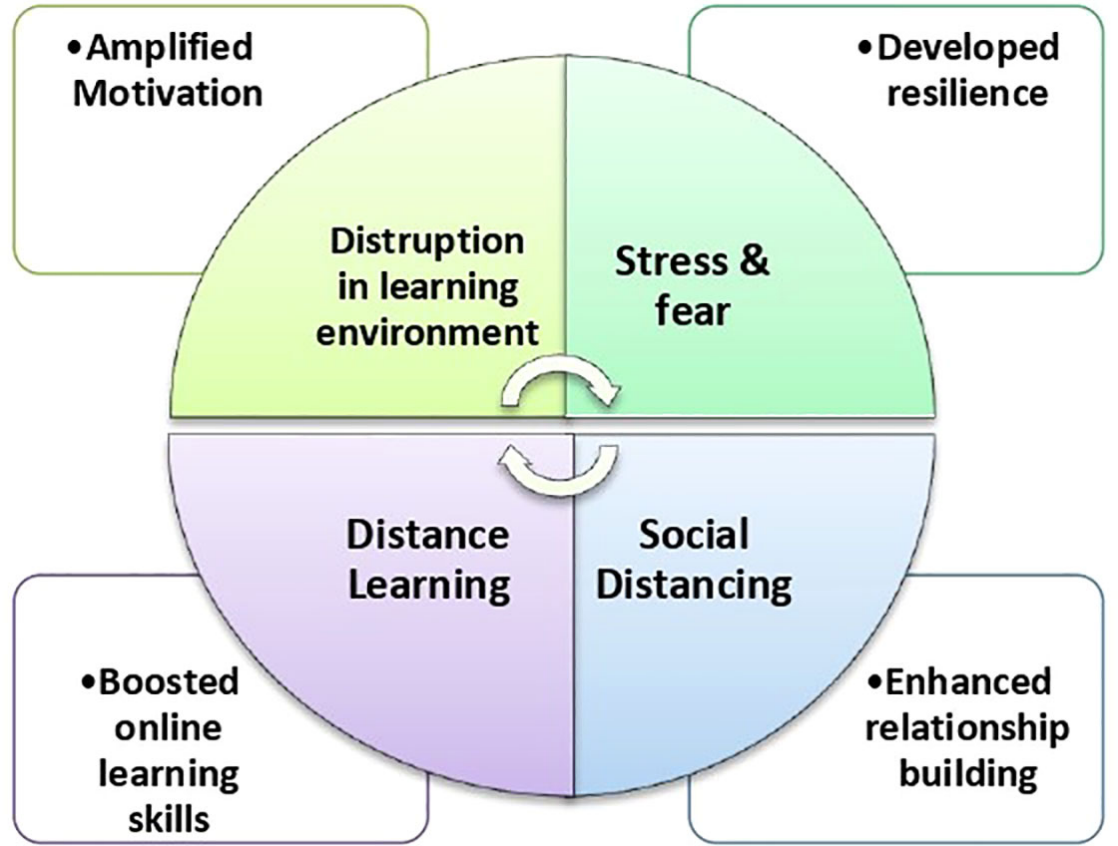
Impact of change agent on the development of key traits and skills among medical student peer learning among medical students during COVID-19 Pandemic

A famous writer Joseph Joubert was true in his vision when he uttered the golden words, “To teach is to learn twice”. In peer learning groups, it was an opportunity to enhance our communication skills through group discussions on decided topics ( Gelis *et al.*, 2020). Communication skill is of utmost importance in the workplace because one has to set the tone for how people perceive you and your ideas during teamwork. Our motto was “you teach me, I teach you”. Therefore, we had a suave transition from on-campus to online learning. The moral support with pair and shared approach encouraged the smooth adaptability to a new learning paradigm. Figure 1 summarises the impact of peer learning benefits during the COVID-19 crisis. It high lights the impact of change agents on the development of key traits and skills among medical students through peer learning strategies.

### Impact on Motivation

One of the major advantages of peer learning during COVID-19 was keeping the morale high by motivating each other. As the ongoing situation was very tough to modify the schedule, weekly timetable amendments, change in the mode of assessment (online exams). Our peer groups motivated and morally supported each other. It was convenient to understand each other’s emotions and levels of anxiety as all of us were sailing the same boat. We focused on learning objectives and worked on the philosophy “Alone we can do so little; together we can do so much.”

This was a marvellous opportunity for group leaders to polish leadership qualities ( Szteinberg *et al.*, 2020). This way of learning stimulated us to enhance our emotional intelligence, discipline, and integrity among group members. We developed more confidence to speak during informal discussions that empowered our self-esteem. Building a safe and friendly environment pushed us to generate active discussion on the topics (clinical scenarios). Eventually, we could spend more time discussing the depth and width of topics; leading to deep learning.

### Stress management

Experiencing anxiety during a pandemic is rational, but sometimes it is overblown depending upon one’s personality traits. The pandemic caused significant anxiety among medical students in the initial phase ( Jin, Wang, and Lan, 2019). Among vulnerable students, there was continuous stress, and anxiety led to a pounding heart, sleep disturbance, a change in eating habits, impaired performance, and depression. However, through peer learning style students shared thoughts and emotional reactions freely and friendly. This shared approach assisted us to measure the level of anxiety and aided to find more emotionally vulnerable and fragile personalities.

We supported each other affectionately and avoided engaging in the infodemic of COVID-19. We commended each other to have minimum information about morbidities and mortalities around the word. The purpose was to stay informed about the situation but not to stimulate fight and flight response or limbic systems stimulation, which causes extreme anxiety. We urged each other to focus on our studies and stay safe rather than spend our energy on a rapidly changing environment. Few of us experienced mindful body practice to train the mind for maximum focus and stress management strategy ( Crowther, Robertson and Anderson, 2020).

We motivated each other to energise our body by keeping healthy habits, for instance, eating a healthy diet which is important for the plasticity in the brain. We encouraged each other to take slight breaks and eat small snake during the planned study session. Besides, we stimulated each other for online virtual exercise practices to boost immunity. We encouraged normal sleep hours and reduce screen time on phones to avoid fatigue due to blue rays and sickness of computer screens. Coping with stress through peer learning style developed resilience among students and helped to come out of the paralyzed state of anxiety ( Walsh *et al.*, 2020).

### Online learning skills (Technology-enhanced learning)

This pandemic is teaching all of us in different dimensions and how to cope with our circumstances. As it switched all teaching activities to technology-enhanced learning, we found ourselves in the beginning zone of a hundred percent online. We needed technical support to get used to the learning management system. Peer learning helped us to adapt to technology-enhanced learning more smoothly and effectively. If we faced any issue in soft skills, we seek help from each other promptly. Most of us recognised the improvement in the online teaching skills through peer learning style.

### Improving students’ social interactive learning

Although there were instructions by health care professionals to keep social distance during the COVID-19 crisis, peer learning encouraged us to engage more deeply with each other. Through this way of informal discussion, we had closer social interaction virtually. Although we had been together for five years in the same class, we had never got to know about our peers before. We encountered various behaviours and learning attitudes. It was a natural opportunity to develop a friendly relationship with our peers. This approach helped students who live in a different region and away from the home city. Saying connected with our peers during the online sessions augmented the ability to combat the crisis.

## Challenges

The effectiveness of peer learning needs to be investigated through qualitative and quantitative studies. It will help us to establish the authenticity of the peer learning strategy during the adaption process to a new learning paradigm. Additionally, it is important to enquire about the views of those students who could not adjust in this way of learning. It is imperative to identify the additional factors which hinder adaptation to peer learning. An in-depth analysis is required to determine the barriers and shortcomings of peer learning.

## Conclusion

During the COVID-19 pandemic, medical students are facing substantial challenges in terms of graduation, securing an internship placement, and entry in professional life smoothly. A major disruption in the learning environment, social distancing, fear of disease, and uncertainty about the future have a great impact on learning. Adaptation to peer learning strategy may help to overcome this stressful situation and motivate each other to focus on studies. This approach can assist medical students to stay in touch with each other, collaborate, communicate, and boost each other morally.

## Take Home Messages

• Peer learning improves the learning process through active discussion, sharing ideas, and encourages students toward higher-level thinking.
• During the COVID-19 crisis, peer learning is a very effective learning approach by motivating each other.
• The peer learning style provides an opportunity for students to share thoughts and emotional reactions freely and friendly. This way can help to reduce stress and develop resilience.
• Students get fast adaptation to technology-enhanced learning more smoothly and effectively by helping each other to learn new skills.
• The feeling of staying connected with peers during the online sessions significantly augmented the ability to combat the crisis and augment social interactions.

## Notes On Contributors

**Sara Mohammed Sami Hamad** is a final year medical student in Alfarabi College of Medicine, Riyadh and main author of this manuscript.

**Dr Shazia Iqbal** is working as Assistant Professor/Director of Medical Education Unit, Clinical faculty in Alfarabi College of Medicine, Riyadh, Saudi Arabia. She is keenly involved to recognize gaps in medical education with a special interest in pedagogical techniques and technology enhanced learning. She is co-author of this manuscript. ORCID ID: https://orcid.org/0000-0003-4890-5864

**Alreuof Mohammed Alothri** is final year medical student in Alfarabi College of Medicine, Riyadh. She was involved in literature review and writing references.

**Manal Abdullah Ali Alghamadi** is final year medical student in Alfarabi College of Medicine, Riyadh. She was involved in editing and reviewing this manuscript.

**Manal Khalid Kamal Ali Elhelow** is final year medical student in Alfarabi College of Medicine, Riyadh. She is a representative of peer group leader and gathered information from groups to complete this piece of opinion.
