# Supplementary material for: “To teach is to learn twice” Added value of peer learning among medical students during COVID-19 Pandemic
Source: MedEdPublish (2016). 2021 Jun 29;9:127. Originally published 2020 Jun 22. [Version 2] doi: 10.15694/mep.2020.000127.2 (PMC10702662; doi:10.15694/mep.2020.000127.2)
Supplement: Peer review history

# Reviewer report: Reviewer response for version 2

**DOI:** 10.21956/mep.20269.r31462

**Published:** 2021-10-08

**Authors:** Julie Hunt

**Affiliations:** 1 Lincoln Memorial University

This review has been migrated. The reviewer awarded 3 stars out of 5 I commend this group of student authors for their contribution. The pandemic has been a challenging period of time for both educators and students, and the perspective of students is helpful to educators. I would have preferred that the authors include the year when listing dates, as their publication could be read or cited into the future, and this is clearer to readers. The article would have been stronger if the authors had spent some time describing the peer learning groups. Were they chosen or assigned? How large were the peer groups? Were the same peer groups used for all subjects, or did groups shift for different subjects? Were the peer groups the same for an entire semester, or was there any shifting?The authors comment on their improved performance on formative assessments and online written assessments as compared with summative assessments, but I wonder, did that occur because formative assessments and written assessments might be easier than the previous summative assessments? More detail is needed to allow readers to understand how and why test performance was improved.Finally, the authors included only a single sentence in the challenges section to mention that there were students who didn't adapt easily to the new learning methods. Many educators have observed that there are students who have not flourished in the new e-learning period. As the entire article is very favorable to the transition, I think it is important to discuss also that there are students who have not adapted, and why that may have occurred, how it was addressed (if at all), etc.Overall it is refreshing to hear students' voices explaining some of the beneficial adaptations made during the pandemic.

Reviewer Expertise:

NA

No decision status is available

# Reviewer report: Reviewer response for version 2

**DOI:** 10.21956/mep.20269.r31461

**Published:** 2021-06-29

**Authors:** Ronald M Harden

**Affiliations:** 1 AMEE

This review has been migrated. The reviewer awarded 4 stars out of 5 Student engagement with students as partners in the learning process is high on today's agenda in medical education. In this paper the authors provide a student perspective. They describe in the context of the Covid pandemic the perceived benefits with reference to the literature. Although there is no objective evidence given, the students' views are convincing and I recommend you to read this article. There are places where the text could be improved and the occasional mistake such " eat small snake" rather than " small snack". The reference to the literature is not extensive and no mention for example of Tippings classic text - Effective peer learning. This article is however a recommended read.

Reviewer Expertise:

NA

No decision status is available

# Reviewer report: Reviewer response for version 1

**DOI:** 10.21956/mep.19135.r27641

**Published:** 2020-07-27

**Authors:** Mildred López

**Affiliations:** 1 Tecnologico de Monterrey, School of Medicine and Health Sciences

This review has been migrated. The reviewer awarded 3 stars out of 5 First, I would like to congratulate the students on getting involved in medical education and sharing their personal views on a manuscript. I also would like to recognize the educators and research groups that support those practices.The manuscript presents broad concepts: “peer-learning” and “traits and skills”, but it needs to explain the connection on the impact on traits and skills, developed through peer-learning. I believe it is not written as an opinion piece. At the beginning, it starts as if it was going to describe an innovation implementation of peer-learning. However, it lacks the documentation or assessment to analyze the “added value” of the experience. The authors should commit to which approach to take:1) the “opinion piece” of their learning of this pandemic. The authors could revise the manuscript and focus on describing how final-year students experienced the distance education transition during the COVID19, or 2) describe the experience of peer-learning during the pandemic. It could be that the contributions that they perceive, are presented in a model (such as the one presented in figure 1). I think that more than presenting it as a cycle, it should be a table that relates each distance education element to a benefit/advantage perceived by the students. These claims should also be supported by literature and examples given by students.I would encourage the authors on revising the manuscript and upload a version that integrates the comments made by other reviewers.

Reviewer Expertise:

NA

No decision status is available

# Reviewer report: Reviewer response for version 1

**DOI:** 10.21956/mep.19135.r27638

**Published:** 2020-07-26

**Authors:** P Ravi Shankar

**Affiliations:** 1 American International Medical University

This review has been migrated. The reviewer awarded 4 stars out of 5 This is an important article on peer learning and support during the significant disruption caused by the COVID-19 pandemic. I am happy to note that most of the authors are medical students. The authors describe what was done clearly. I am not sure about the use of the term ‘peer learning style’ as learning styles have a specific connotation. The authors can provide more information about the final year medical students at the university. What is the student strength? Are there international students? The authors mention about both student pairs and student groups. As the groups were self-selected, I assume those who knew each other well formed pairs and groups. The process of group formation can be described in greater detail. I assume that this is a college only for women and all groups consisted only of females. This is an important point to confirm as gender could influence relationships and group dynamics. The advantages of peer learning have been well described. Were there any challenges? The stress students were going through can affect groups and group dynamics? Did faculty members have any role with regard to group formation and maintenance? In certain areas of the manuscript language and grammar corrections can be carried out.

Reviewer Expertise:

NA

No decision status is available

# Reviewer report: Reviewer response for version 1

**DOI:** 10.21956/mep.19135.r27637

**Published:** 2020-07-07

**Authors:** Poh-Sun Goh

**Affiliations:** 1 National University of Singapore

This review has been migrated. The reviewer awarded 3 stars out of 5 Thank you to the authors for nominating me to review this paper. I reviewed this paper with interest and anticipation, as it represents, and gives visibility and a voice to the perspectives of medical students, who are the future healthcare practitioners that we as educators strive to support, assist, guide and instruct.The key messages, as well as proportion and percentage of text given to each one of the key messages was particularly striking. The title, and first sentence of the abstract alludes to deeper learning, and more effective learning as the major impact of peer learning during the COVID-19 pandemic; while the bulk of the paper focused on the human side of interpersonal interactions, providing motivation and support to engage in learning activities, with an outcome of better assessment performance being briefly mentioned during April exams. I feel this paper would be significantly improved by providing more information, for example - 1) more background information about the setting; 2) how the COVID-19 pandemic disrupted previous practice; 3) how the reflective exercise was performed with the students, and much more detailed breakdown on participation rates, qualitative and quantitative responses, and how the reflective exercise was facilitated, and how the data/responses were analysed; including whether further iterative follow up engagement with the student cohort was undertaken; 4) more details on how exam performance was improved, and whether this was due in fact to deeper learning, and how peer learning assisted this - was this merely by improved motivation, or deeper knowledge and skill learning, and a reflection and analysis of how and why this took place; 5) more details on how peer learning was felt to improve and deepen knowledge and skill learning, to supplement the bulk of the paper focused on emotional and social support.I look forward to engaging with the authors on this discussion platform, and reading an updated version of this paper in the future.

Reviewer Expertise:

NA

No decision status is available

# Reviewer report: Reviewer response for version 1

**DOI:** 10.21956/mep.19135.r27639

**Published:** 2020-07-01

**Authors:** sathyanarayanan varadarajan

**Affiliations:** 1 SRM Medical College, SRM University

This review has been migrated. The reviewer awarded 4 stars out of 5 This interesting article by a final year medical student highlights the adjustment of the final year medical students to the peer learning style during the COVID-19 pandemic. The authors have explored nicely on the learning experience of adapting peer learning strategies and various benefits of adopting peer learning in this rapidly changing environment. One significant observation they made during peer learning process was that the students felt staying connected with peers during the online sessions, which significantly augmented the ability to combat the crisis and augment social interactions. They also felt motivated and it helped them to adopt to technology-enhanced learning more smoothly and effectively. I congratulate the author for her writing style, expressing complex things in simple words. Though the effectiveness of peer learning needs to be investigated through qualitative and quantitative studies, this article makes an important contribution in the field of new learning and teaching strategies, in this significant transition period.

Reviewer Expertise:

NA

No decision status is available

# Reviewer report: Reviewer response for version 1

**DOI:** 10.21956/mep.19135.r27640

**Published:** 2020-06-25

**Authors:** Shahzad Ahmad

**Affiliations:** 1 Fatima Memorial medical and dental college Lahore Pakistan

This review has been migrated. The reviewer awarded 5 stars out of 5 I enjoyed reading this paper consisting of the reflection of the medical students regarding their approach to peer learning during COVID-19. The manuscript has been well written and we need to hear the voice of medical students during current situation. The reflection highlights the natural adoption to critical situation and brings the new version of peer learning (pedagogical techniques) in medical education. It explored the extraordinary benefits of peer learning such as learning of new technology skills; powerful impact on motivating each other and enhancement of inter-professional relation despite of social distancing during COVID-19. For better adoption to current situation, the given recommendation can be applied as unique way of learning (peer learning) in medical education. I do think that this paper should be read by all medical students, faculty and medical educators.

Reviewer Expertise:

NA

No decision status is available
